# Community-acquired pseudomonas keratitis: an unusual presentation in a 2-month old infant that led to corneal transplant

**DOI:** 10.5935/0004-2749.20220091

**Published:** 2025-08-21

**Authors:** Fernando Henrique Flores Teixeira, Ana Luiza Biancardi, Monick Goecking Cardoso Vieira, Julia Alves, Nycholas Tavares, Paulo Phillipe Moreira, André Luiz Land Curi

**Affiliations:** 1 Laboratory of Infectious Ophthalmology, Fundação Oswaldo Cruz, Rio de Janeiro, RJ, Brazil; 2 Instituto de Puericultura e Pediatria Martagão Gesteira, Universidade Federal do Rio de Janeiro, Rio de Janeiro, RJ, Brazil; 3 Centro de Estudos e Pesquisas Oculistas Associados, Rio de Janeiro, RJ, Brazil

Dear Editor,

Corneal disease remains the leading cause of monocular blindness worldwide, especially affecting marginalized populations. Corneal opacities, which are largely caused by infectious keratitis, are the fourth leading cause of blindness globally and responsible for 10% of avoidable visual impairment in the world’s least developed countries^(1)^. Herein, we report a case of aggressive infectious keratitis caused by *Pseudomonas aeruginosa* in an otherwise healthy 2-month-old infant with no previous risk factors of ocular infections, which led to corneal transplant.

A healthy 2-month-old female infant arrived at the *Instituto de Puericultura e Pediatria Martagão Gesteira* presenting with a 2-day history of conjunctival hyperemia, chemosis, purulent exudation, and diffuse corneal opacity in the left eye (OS). The right eye (OD) examination result was normal. She received tobramycin eye drops for 24 h, which was prescribed by a general pediatrician in the emergency department, but this brought no improvement. Her past medical and ocular histories were unremarkable. The mother had preeclampsia and gestational diabetes during pregnancy. She denied sexually transmitted diseases and had negative serological test results for HIV, hepatitis B, syphilis, and toxoplasmosis. The baby was born via vaginal delivery at 38 weeks of pregnancy and was delivered 2 hours after membrane rupture, without any complications. She was discharged from the hospital 48 h after birth, with negative results in all screening tests.

**Figure 1:**
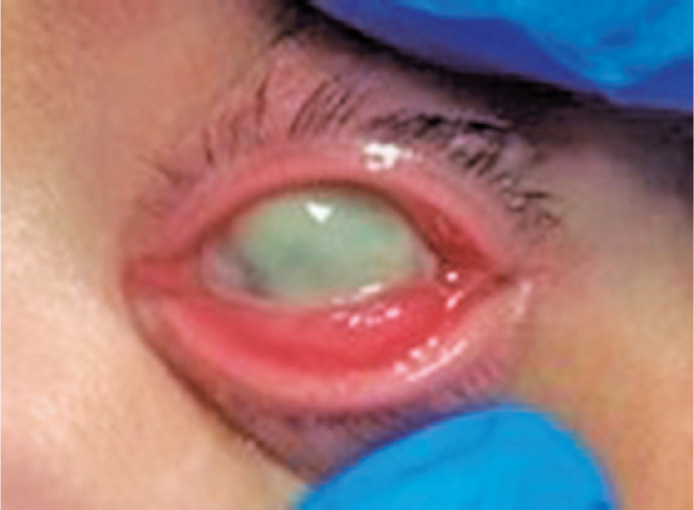
The patient presenting with conjunctival hyperemia, chemosis, corneal infiltrate, and melting.

The patient was hospitalized, and swab specimens were collected from ocular secretions. On the following day, she presented a hypopyon that covered three-fourths of the corneal diameter and corneal melting (Figure 1). The eye drops were then switched from tobramycin to moxifloxacin.

Two days after hospitalization, the patient evolved to corneal perforation and uveal tissue tamponade. The eye drops were again switched to fortified vancomycin, ceftazidime, and tobramycin. Intravenous azithromycin was added to reduce the corneal melting. The infant was also scheduled for corneal transplantation.Five days after hospital admission, the cultures of the ocular secretions were negative for methicillin-resistant *Staphylococcus aureus* (MRSA) and positive for *Pseudomonas aeruginosa.* Both the fortified eye drops and intravenous azithromycin therapy were continued.

**Figure 2:**
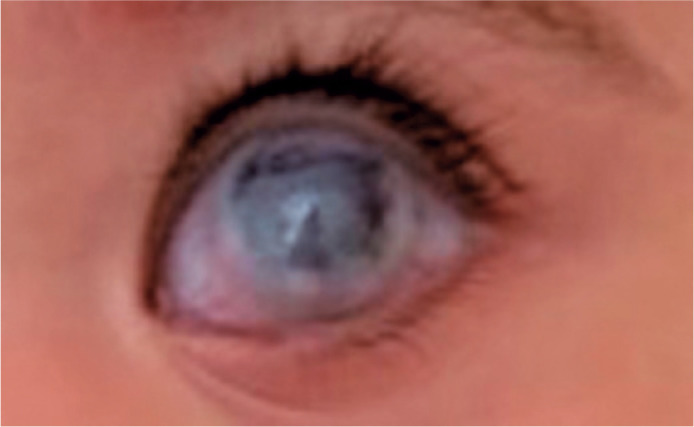
Late postoperative care of corneal transplant.

Despite the aggressive topical and intravenous antimicrobial treatment, after 9 days of hospitalization, the progressive corneal melting resulted in perforation, which led to a successful corneal transplant. At the beginning of the procedure, the lens had spontaneous extrusion through the perforated cornea. The fortified eye drops were continued along with prednisolone during postoperative care (Figure 2), and the patient had a satisfactory anatomical result.

Pediatric bacterial keratitis is an uncommon but potentially devastating condition. It is usually associated with trauma and preexisting corneal disease^(1,2)^, none of which were presented by the proband. The most common causative organisms of bacterial keratitis in the general population are *P. aeruginosa*, coagulase-negative staphylococci, and *Staphylococcus aureus*^(2)^. In one meta-analysis, the prevalence of *P. aeruginosa* isolates in bacterial keratitis ranged from 6.8% to 55%, being strongly associated with contact lens wearing^(3)^. As for the pediatric population, the prevalence of the same pathogen ranges from 10.2% to 20%^(1)^.

Empirical treatment of microbial keratitis in children consists of combined fortified antibiotics in most cases (51.4%), with the most frequent combination being tobramycin and vancomycin^(3)^. Treatment with a single agent such as fluoroquinolone is also a common approach^(3)^. The present patient was initially treated by the ophthalmologic medical team with moxifloxacin eye drops, which has been shown to have comparable efficacy with fortified antibiotics for the management of bacterial keratitis^(4)^.

In such cases, when fluoroquinolone eye drops are chosen as an initial approach, assessment of the response to treatment is critical because if the patient shows worsening of symptoms, one can consider switching to fortified broad-spectrum antibiotics, which happened on the following day in the present case. Intravenous azithromycin was also used to reduce corneal melting and prevent perforation, given that the effects of macrolide on corneal epithelial cell apoptosis may promote epithelial healing, which can subsequently lead to a decrease in stromal degradation^(5)^.

In conclusion, infectious keratitis is an important cause of childhood vision loss, and timely diagnosis and treatment are paramount to improve the visual prognosis. Both pediatricians and ophthalmologists should be aware of its signs and symptoms, and the challenges and updates of its management to prevent amblyopia and blindness.
